# Unhealthy Gambling Amongst New Zealand Secondary School Students: An Exploration of Risk and Protective Factors

**DOI:** 10.1007/s11469-015-9562-1

**Published:** 2015-06-30

**Authors:** Fiona V. Rossen, Terryann Clark, Simon J. Denny, Theresa M. Fleming, Roshini Peiris-John, Elizabeth Robinson, Mathijs F. G. Lucassen

**Affiliations:** Social and Community Health, School of Population Health, Faculty of Medical and Health Sciences, University of Auckland, Private Bag 92019, Auckland, 1142 New Zealand; School of Nursing, University of Auckland, Auckland, New Zealand; Department of Paediatrics: Child and Youth Health, University of Auckland, Auckland, New Zealand; Department of Paediatrics: Child and Youth Health & Department of Psychological Medicine, University of Auckland, Auckland, New Zealand; Section of Epidemiology and Biostatistics, University of Auckland, Auckland, New Zealand; Auckland UniServices Ltd, Auckland, New Zealand; Department of Psychological Medicine, University of Auckland, Auckland, New Zealand

**Keywords:** Youth, Gambling, Risk factors, Protective factors, Mental Health

## Abstract

This study sought to determine the prevalence of gambling and unhealthy gambling behaviour and describe risk and protective factors associated with these behaviours amongst a nationally representative sample of New Zealand secondary school students (*n* = 8,500). Factor analysis and item response theory were used to develop a model to provide a measure of ‘unhealthy gambling’. Logistic regressions and multiple logistic regression models were used to investigate associations between unhealthy gambling behaviour and selected outcomes. Approximately one-quarter (24.2 %) of students had gambled in the last year, and 4.8 % had two or more indicators of unhealthy gambling. Multivariate analyses found that unhealthy gambling was associated with four main factors: more accepting attitudes towards gambling (*p* < 0.0001); gambling via gambling machines/casinos/track betting (*p* = 0.0061); being worried about and/or trying to cut down on gambling (*p* < 0.0001); and, having attempted suicide (*p* = 0.0009). Unhealthy gambling is a significant health issue for young people in New Zealand. Ethnic and social inequalities were apparent and these disparities need to be addressed.

## Introduction

Gambling is a widely available activity in industrialised societies; and an entire generation has grown up in an era when lottery and casino gambling is widely available and heavily advertised (Volberg, Gupta, Griffiths, Ólason, and Delfabbro 2010). Gambling has therefore become a popular pastime not only among adults, but also among children and young people (Derevensky and Gupta 2000; Splevins, Mireskandari, Clayton, and Blaszczynski 2010) with gambling being one of the first ‘risky activities’ that adolescents engage in, even prior to experimenting with alcohol and drugs, or engaging in sexual behaviour (Volberg et al. 2010). Youth gamblers engage in a range of gambling activities, from informal modes such as betting amongst friends, through to more formal activities such as ‘fruit machines’, electronic gambling machines (EGMs) and lottery-based products (Department of Internal Affairs 2008; Felsher, Derevensky, and Gupta 2003; Ipsos MORI, 2012; Stinchfield 2001; Turner, Macdonald, Bartoshuk, and Zangeneh 2008). Research has shown that access to this latter type of gambling remains accessible to young people, despite age-related restrictions (Department of Internal Affairs 2008; Fisher 1999; Rossen, Butler, and Denny 2011; Rossen, Tse, and Vaidya 2009). Research also suggests that gambling behaviour may change as young people progress developmentally, with accessible (but age-restricted) activities such as lottery products and EGMs becoming more attractive to older adolescents (Rossen et al. 2009; Stinchfield 2001; Volberg et al. 2010). It is worth noting that in New Zealand the legal age for gambling varies according to the specific activity, in particular: 20 years of age for casino gambling; and, 18 years of age for Instant Kiwi (scratch tickets), TAB (track and sports) betting and EGMs in pubs or clubs. With the exception of Instant Kiwi, there are currently no age limits on the various lottery products provided by Lotto New Zealand (a Crown Entity that was established in 1987).

Whilst for many young people involvement in gambling does not appear to have negative impacts on their health and wellbeing, research globally has documented that a sub-set of youth go on to experience serious problems (Hardoon and Derevensky 2002; Huang and Boyer 2007; Jackson, Dowling, Thomas, Bond, and Patton 2008; Turchi and Derevensky 2006; Volberg et al. 2010; Welte, Barnes, Tidwell, and Hoffman 2008) with those who start gambling at an earlier age more likely to develop more severe gambling problems (Huxley and Carroll 1992; Volberg, Abbott, Ronnberg, and Munck 2001). A recent review estimates that 4–8 % of young people gamble at problem/pathological levels with a further 10–15 % at risk of developing a gambling problem (Volberg et al. 2010). Rates of unhealthy gambling among young people is higher than that identified for adults, with some estimates of youth rates being at least double those of adults (Volberg et al. 2010).

The social and other costs of problem gambling experienced by young people have been widely reported in the literature. In particular, the co-existence of problem gambling and poor mental health has been noted, with depression and suicide attempts frequently associated with youth gambling (Derevensky and Gupta 2000; Volberg et al. 2010). The co-existence of problem gambling and engagement in other addictive behaviours has also been documented. For example, substance misuse is a significant risk factor for unhealthy youth gambling (Dickson, Derevensky, and Gupta 2008; Fisher 1999; Goldstein et al. 2013; Griffiths and Wood 2000; Moodie and Finnigan 2006; Rossen et al. 2011; Stinchfield 2000), with alcohol use being one of the strongest predictors of gambling amongst high school students in the United States (Stinchfield 2000). Disruptions to family relationships and other relationships, as a result of gambling, have also been reported in the literature (Blinn-Pike, Worthy, and Jonkman 2010; Fisher 1999; Floros, Siomos, Fisoun, and Geroukalis 2013; Hardoon, Derevensky, and Gupta 2002; Hardoon and Derevensky 2002; Splevins et al. 2010). Furthermore, as gambling progresses into problematic levels, friendships and relationships with non-gambling peers may be replaced by gambling-related associates (Blinn-Pike et al. 2010; Fortune et al. 2013; Gupta and Derevensky 2000). As with other addictive behaviours, when a young person’s gambling behaviour intensifies or becomes problematic, the young person may become increasingly socially isolated (Hardoon and Derevensky 2002; Splevins et al. 2010). Problem gambling amongst youth has also been associated with: delinquency and crime (Blinn-Pike et al. 2010; Clark and Walker 2009; Fortune et al. 2013; Gupta and Derevensky 2000; Valentine 2008); school-related difficulties (e.g. truancy and reduced academic performance) (Blinn-Pike et al. 2010; Dickson et al. 2008; Floros et al. 2015; Splevins et al. 2010); and, financial difficulties (Fisher 1999; Gupta and Derevensky 2000).

Despite previous research on risk factors associated with unhealthy gambling from overseas, to the best of the authors’ knowledge no analysis of risk and protective factors of unhealthy gambling, based on data from population-based studies of adolescents in Australasia, is available in the peer-reviewed literature.

This study aimed to report the prevalence of unhealthy gambling behaviours and describe risk and protective factors associated with unhealthy gambling behaviours, amongst a recent and large nationally representative sample of high school students in New Zealand. A comprehensive understanding of the risk and protective factors associated with adolescent gambling will assist in health promotion efforts to improve awareness of youth gambling issues and related help-seeking behaviours. This is particularly important as young people tend to seek help for gambling-related problems from informal sources, such as friends and their family, rather than from professional healthcare providers (Griffiths 2001; Gupta and Derevensky 2000).

## Methods

This study used data collected as part of Youth’12, the third national health and wellbeing survey of secondary school students in New Zealand conducted in 2012. A detailed description of the Youth’12 survey methodology is available elsewhere (Clark et al. 2013). In summary, 8,500 randomly selected secondary school students throughout New Zealand took part in Youth’12. In total, 91 randomly selected high schools participated in the survey, accounting for 3 % of the total 2012 secondary school roll in New Zealand. Response rates for schools and students were 73 % and 68 % respectively. The comprehensive 608-question survey was anonymous and administered via internet tablets (Denny et al. 2008). Written consent was required from each participating school and each student prior to participation. The University of Auckland Human Participants Ethics Committee approved the study.

## Measures

Participants were asked “What sex are you?” (response options were “Male” or “Female”) and they were asked to indicate their age in years. Due to small numbers for some analyses, age was categorised as ‘15 or less’ and ‘16 or older’. This categorisation was chosen as it is consistent with the age that New Zealand adolescents can leave school (i.e. students can leave school at 16 years of age). Students indicated the ethnic group(s) that they belonged to. Response options were based on the New Zealand Census standard 2001/2006 ethnicity questions (Statistics New Zealand 2005) and participants who chose more than one ethnicity were assigned a single ethnic group based on the Statistics New Zealand ethnicity prioritization method (Lang 2002). Therefore for the present analyses ethnicity was grouped as: Māori, Pacific, Asian, “Other”, and New Zealand European. Neighbourhood socio-economic deprivation was measured using the New Zealand Deprivation Index (NZDep2006) (Salmond, Crampton, Sutton, and Atkinson 2006 ) for the census area unit that the student lived in. NZDep2006 combines eight dimensions of deprivation derived from the NZ census (Salmond et al. 2006 ). For data analyses students were grouped into one of three deprivation bands – lower deprivation (NZ census deprivation deciles 1 to 3), medium deprivation (deciles 4 to 7) and higher deprivation (deciles 8 to 10). A student’s geography was divided into two categories, urban and rural.

For the purpose of this study, gambling was defined as having “bet precious things for money on an activity”. Involvement in gambling was assessed by the question “Have you ever gambled or bet precious things for money on any of these activities?” with the following activities: “Instant Kiwi (scratchies)”; “Lotto (including Strike, Powerball and Big Wednesday)”; “Bingo or Housie”; “Pub or club pokies”; “A casino (e.g. roulette, pokies)”; “TAB betting (e.g. on track racing or sports)”; “Games and gambling on a cell/mobile phone for money or prizes (e.g. txt games)”; “Gambling on the Internet for money or prizes (e.g. internet casinos or poker)”; “Bets with friends or family”; “0900 phone games”; “Cards or coin games (e.g. poker)”. Response options to these items included: “Never”; “Not in the past 12 months”; “Once or twice in the past 12 months”; “Once in the last 4 weeks”; “Two or three times in the last 4 weeks”; “About once a week”; “Several times a week”; and, “Most days”.

Youth’12 is a comprehensive survey (with up to 608 questions) focused on youth health and wellbeing and as such it covers a wide range of domains or topics. While the survey aimed to include as many important questions as possible, we aimed to create a survey that could be completed within approximately one hour, thus preventing participant fatigue. As such, a validated problem gambling screen (e.g. the DSM-IV-MR-J) was not included in Youth’12. The absence of a problem gambling screen resulted in the authors developing a specific model to provide a measure of problematic or unhealthy gambling. This model was developed through an analysis of the previous national youth survey – Youth’07, where factor analysis was used to assess dimensionality of gambling behaviour and the number of underlying factors (Rossen et al. 2011). The relationship between a participant’s responses to items and their level on the underlying latent gambling continuum were examined using two-parameter logistic item-response theory models. Item Response Theory (IRT) was used to model the probability of gambling behaviours along a latent dimension of ‘less unhealthy’ to ‘more unhealthy’ gambling behaviours. This allowed the development of a framework for evaluating which behaviours were more severe. The model utilised seven indicators of unhealthy gambling, including four reasons for gambling that are consistent with escapism and/or loss of control (“I gamble to relax”; “I gamble to feel better about myself”; “I gamble to forget about things”; “I gamble because I can’t stop”) and higher levels of engagement with or expenditure on gambling (gambling ‘several times a week’ or ‘most days’; spending $20 or more per week on gambling; and, spending one or more hours per day on gambling activities). Analyses indicated that it would be appropriate to utilise this model to provide a measure of ‘unhealthy gambling’ for the present study. Students were classified as having ‘none’, ‘one’ or ‘two or more’ indicators of unhealthy gambling. A full description of the development of this measure can be found in the associated Youth’07 report (Rossen et al. 2011). A description for all the outcome variables and measures that were utilised in this study are available from the first author upon request (of note, these variables were based on a comprehensive review of the gambling literature and consultation with an expert advisory group that guided the overall research project) (Rossen et al. 2013).

## Analyses

All statistical analyses have accounted for the sample design and clustering effects within schools; data have been weighted by the inverse probability of selection and the variance of estimates were adjusted to allow for correlated data from the same schools. Total numbers and prevalence estimates, adjusted percentages, and 95 % confidence intervals were calculated for the selected outcomes. Logistic regression and multiple logistic regression models were used to investigate the associations between unhealthy gambling behaviour and outcomes. Logistic regression models included the possible confounders of age, sex, ethnicity, urban/rural status and level of neighbourhood socioeconomic deprivation. All statistical analyses were carried out using SAS software (version 9.3) (SAS Institute Inc 2011). Given the sample size and number of comparisons, a p-value of ≤0.01 was taken to indicate statistical significance.

## Results

### Involvement in Gambling

One-in-10 students had gambled in the last 4 weeks and almost one-quarter (24.2 %) had gambled in the last year (see Table 1). Overall, rates of gambling were spread across demographic variables such as age group, urban/rural setting, and level of neighbourhood deprivation. However, significantly greater proportions of males than females had gambled in the last 4 weeks and in the last year. There was also a significant association between ethnicity and gambling in the last 4 weeks with the non-New Zealand European students being proportionately more likely to gamble, and this was especially so for Māori and Pacific students.

**Table 1:** Gambling in the past month and past year by demographic features

	Gambled in the last 4 weeks			Gambled in the last 12 months ^1^		
	n (N)	% (95 % CI)	p-value	n (N)	% (95 % CI)	p-value
Total	804 (7,813)	10.3 (9.4–11.3)		1890 (7,813)	24.2 (23.1–25.3)	
Sex						
Male	440 (3,456)	12.8 (11.7–13.8)	<.0001	911 (3,456)	26.4 (24.7–28.1)	<.0001
Female	363 (4,355)	8.4 (7.4–9.3)		978 (4,355)	22.5 (21.4–23.6)	
Age						
15 or less	529 (5,033)	10.6 (9.4–11.7)	0.6462	1,189 (5,033)	23.7 (22.4–24.9)	0.09
16 or more	273 (2,770)	9.8 (8.6–11.1)		697 (2,770)	25.2 (23.3–27.1)	
Ethnicity						
Māori	180 (1,490)	12.1 (10.4–13.8)	0.0008	375 (1,490)	25.1 (22.7–27.5)	0.08
Pacific	142 (1,007)	14.2 (10.8–17.5)		264 (1,007)	26.3 (23.2–29.3)	
Asian	91 (983)	9.3 (7.9–10.8)		221 (983)	22.6 (20.0–25.1)	
Other	55 (474)	11.6 (8.5–14.7)		113 (474)	23.9 (19.5–28.3)	
NZ European	335 (3,852)	8.7 (7.5–9.9)		916 (3,852)	23.8 (22.3–25.4)	
NZDep2006						
Lower	239 (2,598)	9.3 (7.8–10.7)	0.3063	639 (2,598)	24.7 (22.6–26.7)	0.52
Medium	272 (2,809)	9.7 (8.5–10.9)		667 (2,809)	23.8 (22.1–25.4)	
Higher	286 (2,327)	12.4 (10.5–14.2)		565 (2,327)	24.4 (22.5–26.3)	
Geography						
Urban	693 (6,563)	10.6 (9.6–11.6)	0.3121	1,580 (6,563)	24.1 (23.0–25.3)	0.54
Rural	104 (1,171)	8.9 (7.3–10.5)		291 (1,171)	24.8 (22.2–27.5)	

1. The percentages reported here are inclusive of students who had gambled in the last 4 weeks

### Unhealthy Gambling Behaviour

While the majority of students who had gambled in the last 12 months did not report any indicators of ‘unhealthy gambling’ (84.3 %, *n* = 1557), approximately one-tenth (11.0 %, *n* = 203) reported one indicator and approximately five percent (*n* = 89) reported two or more indicators of ‘unhealthy gambling’ (see Table 2). Significant interactions were observed between the distribution of ‘unhealthy gambling’ indicators and sex (males more than females); ethnicity (Māori, Pacific and Asian students more than New Zealand European); and neighbourhood deprivation (living in highly deprived neighbourhoods more than lower deprivation neighbourhoods).

**Table 2:** Distribution of unhealthy gambling by demographic features^a^

	Number of Unhealthy Gambling Indicators ^b^						p-value
	None		One		Two or more		
	n (N)	% (95 % CI)	n (N)	% (95 % CI)	n (N)	% (95 % CI)	
Total	1,557 (1849)	84.3 (81.9–86.7)	203 (1,849)	11.0 (9.2–12.7)	89 (1,849)	4.8 (3.5–6.0)	n/a
Sex							
Male	702 (889)	79.1 (76.1–82.0)	124 (889)	13.9 (11.5–16.3)	63 (889)	7.0 (5.3–8.7)	<.0001
Female	855 (959)	89.2 (86.4–91.9)	79 (959)	8.2 (6.0–10.4)	25 (959)	2.6 (1.4–3.9)	
Age							
15 or less	965 (1,158)	83.4 (80.7–86.1)	134 (1,158)	11.6 (9.5–13.7)	59 (1,158)	5.1 (3.6–6.5)	0.53
16 or more	589 (687)	85.8 (82.5–89.1)	68 (687)	9.9 (7.3–12.4)	30 (687)	4.3 (2.7–6.0)	
Ethnicity							
Māori	290 (367)	79.5 (75.2–83.8)	53 (367)	14.2 (10.7–17.7)	24 (367)	6.3 (3.6–9.0)	<.0001
Pacific	178 (253)	70.4 (66.1–74.7)	50 (253)	19.7 (15.8–23.6)	25 (253)	9.9 (6.5–13.3)	
Asian	177 (217)	81.5 (76.8–86.2)	24 (217)	11.2 (6.6–15.7)	16 (217)	7.4 (3.6–11.1)	
Other	91 (108)	84.5 (77.6–91.4)	10 (108)	9.3 (2.8–15.8)	7 (108)	6.2 (1.8–10.6)	
NZ European	821 (903)	90.9 (89.0–92.8)	66 (903)	7.3 (5.5–9.1)	16 (903)	1.9 (0.7–2.9)	
NZDep2006							
Lower	564 (627)	89.9 (87.5–92.4)	47 (627)	7.5 (5.5–9.6)	16 (627)	2.5 (1.4–3.7)	0.0099
Medium	560 (654)	85.7 (82.9–88.4)	61 (654)	9.3 (7.0–11.7)	33 (654)	5.0 (3.3–6.7)	
Higher	419 (550)	76.3 (72.8–79.8)	92 (550)	16.6 (13.6–19.7)	39 (550)	7.1 (5.0–9.2)	
Geography							
Urban	1,289 (1,547)	83.3 (80.8–85.9)	179 (1,547)	11.6 (9.6–13.5)	79 (1,547)	5.1 (3.7–6.5)	0.41
Rural	254 (284)	89.8 (86.7–93.0)	21 (284)	7.3 (4.5–10.0)	9 (284)	2.9 (1.1–4.7)	

^a^Amongst students who had gambled in the past 12 months

^b^Based on the construct of unhealthy gambling behaviour developed as part of this study

### Identification of Risk and Protective Factors

Table 3 provides results from a series of logistic regression analyses between the measure of ‘unhealthy gambling’ behaviour and each of the hypothesised risk/protective factors (phase one analyses). In total, 24 variables were significantly associated with ‘unhealthy gambling’ behaviour; 22 were associated with an increased risk (e.g. being same/both-sex attracted or not-sure of one’s sexual attractions) and two variables moderated/protected against the risk of ‘unhealthy gambling’ behaviour. In particular, good wellbeing (which was based on the WHO-5 Well-Being Index - a short self-administered questionnaire that covers five-items relating to positive mood, vitality and general interests) (World Health Organization 1998) and family connectedness acted in a protective manner.

**Table 3:** Phase One and Phase Two–results of logistic regression models to identify individual risk and protective factors^a^

Outcome variables (categorical)	Number of Unhealthy Gambling Indicators^b^						Phase ONE		Phase TWO	
	None		One		Two or More		p-value^c^	Risk, protective, non-sig (−)	p-value^c^	Risk, protective, non-sig (−)
	*N* = 1,557	% (95 % CI)	*N* = 203	% (95 % CI)	*N* = 89	% (95 % CI)				
Activities that student has gambled on (last 12 months):										
Lotteries and/or Housie	690	44.3 (42.0–46.6)	95	46.1 (39.8–52.4)	47	52.7 (41.1–64.3)	0.11	–	–	–
EGMs/Casino/TAB betting	135	8.6 (6.9–10.3)	51	25.0 (19.5–30.5)	32	35.5 (26.2–44.8)	<.0001	Risk	0.0061	Risk
Internet or phone betting	89	5.7 (4.3–7.2)	45	22.4 (15.7–29.1)	40	44.7 (33.3–56.1)	<.0001	Risk	0.15	–
Bets with friends or family	1,144	73.4 (71.1–75.8)	163	80.5 (74.8–86.2)	75	84.2 (76.6–91.9)	0.11	–	–	–
Gambling companions:										
Friends	1,013	67.6 (65.0–70.2)	138	71.1 (63.6–78.6)	55	62.6 (53.4–71.8)	0.17	–	–	–
Family	950	63.5 (60.6–66.4)	109	55.9 (48.1–63.8)	45	51.3 (42.7–59.9)	0.15	–	–	–
People they know	49	3.3 (2.2–4.3)	21	10.9 (7.2–14.6)	18	21.0 (12.4–29.6)	<.0001	Risk	0.08	–
People they don’t know	22	1.4 (0.8–2.2)	8	3.9 (0.9–7.0)	12	14.1 (7.0–21.2)	<.0001	Risk	0.0141	–
On their own	103	6.8 (5.3–8.2)	18	9.4 (5.0–13.7)	15	17.1 (9.3–24.9)	0.08	–	–	–
Attitudes and social modelling of gambling:										
More accepting of gambling	632	46.9 (44.1–49.7)	82	49.7 (41.4–58.0)	59	76.3 (67.9–84.8)	<.0001	Risk	<.0001	Risk
Parents gamble	1,257	81.2 (78.9–83.4)	164	81.5 (75.1–87.8)	79	89.1 (82.7–95.4)	0.044	–	–	–
Friends gamble	1,056	68.9 (66.3–71.4)	149	74.4 (68.6–80.1)	68	77.0 (66.6–87.4)	0.020	–	–	–
Concerns about gambling:										
Worried/cut-down	218	14.3 (10.8–17.9)	77	39.2 (31.9–46.4)	50	55.9 (45.1–66.6)	<.0001	Risk	<.0001	Risk
Worried about others	117	7.5 (5.7–9.4)	40	19.7 (14.5–24.9)	31	34.9 (24.5–45.3)	<.0001	Risk	0.16	–
Family impacts of someone else’s gambling:										
Arguments in family	88	5.7 (4.4–7.0)	29	14.6 (9.5–19.7)	22	25.9 (16.2–35.6)	<.0001	Risk	0.72	–
Had to go without	44	2.9 (1.8–3.9)	27	13.5 (8.5–18.4)	23	27.1 (18.1–36.0)	<.0001	Risk	0.79	–
Bills not paid	44	2.9 (2.0–3.8)	26	13.0 (7.8–18.1)	20	23.5 (15.0–32.0)	<.0001	Risk	0.72	–
Dishonest acts	22	1.4 (0.8–2.0)	20	9.9 (5.3–14.5)	20	23.5 (14.9–32.2)	<.0001	Risk	0.16	–
Involvement in other risky behaviours:										
Weekly alcohol use	167	10.7 (8.4–12.4)	19	9.2 (5.1–13.3)	23	25.5 (15.8–35.3)	<.0001	Risk	0.34	–
Binge drinking	449	28.7 (25.8–31.6)	60	29.5 (22.7–36.3)	33	36.8 (25.4–48.2)	0.05	–	–	–
Weekly smoking	65	4.1 (3.2–5.1)	15	7.5 (3.3–11.7)	17	18.8 (11.0–26.7)	<.0001	Risk	0.50	–
Weekly marijuana use	51	3.3 (2.2–4.4)	12	6.0 (3.1–8.9)	12	13.2 (6.7–19.7)	0.01	–	–	–
Emotional health and wellbeing:										
Depression (RADS)	193	12.6 (10.4–14.7)	29	14.7 (10.3–19.1)	25	31.3 (20.0–42.6)	<.0001	Risk	0.22	–
Suicide attempt	55	3.6 (2.5–4.6)	26	12.9 (7.6–18.2)	20	22.0 (13.9–30.2)	<.0001	Risk	0.0009	Risk
Good wellbeing (WHO-5)	1,166	75.2 (72.7–77.7)	149	73.9 (67.8–79.9)	56	63.0 (53.1–73.0)	<.0001	Protective	0.64	–
Violence and bullying:										
Witnessed violence	245	16.1 (13.8–18.5)	63	32.5 (25.8–39.2)	35	41.7 (31.4–51.9)	<.0001	Risk	0.65	–
Victim of violence	205	13.3 (11.5–15.0)	49	25.1 (19.4–30.7)	30	34.9 (25.2–44.5)	<.0001	Risk	0.99	–
Victim of bullying	95	6.1 (4.9–7.2)	18	9.0 (4.3–13.6)	13	14.3 (7.3–21.4)	<.0001	Risk	0.84	–
Sexual attraction:										
Exclusively opposite-sex	1,450	86.2 (84.1–88.3)	170	10.1 (8.4–11.8)	63	3.7 (2.7–4.7)	<.0001	Risk	0.26	–
Same/both sex & not-sure	71	68.9 (59.5–78.3)	19	18.3 (10.7–25.9)	13	12.8 (6.0–19.6)				
Neither sex-attracted	13	49.2 (28.2–70.2)	7	27.4 (10.2–44.5)	6	23.4 (7.6–39.3)				
Other:										
Truancy	373	24.0 (21.5–26.6)	73	35.8 (29.2–42.4)	42	46.5 (35.7–57.2)	<.0001	Risk	0.96	–
Adult support	966	62.3 (59.8–64.8)	119	59.8 (52.3–67.2)	45	52.7 (43.3–62.1)	0.14	–	–	–
Internet use	549	35.6 (32.9–38.3)	94	46.9 (40.5–53.4)	51	58.7 (47.2–70.2)	<.0001	Risk	0.54	–
Computer games	291	18.9 (16.2–21.7)	68	34.0 (27.2–40.8)	41	46.6 (35.9–57.3)	<.0001	Risk	0.35	–
Spiritual beliefs important	342	22.2 (18.1–26.4)	66	33.3 (24.6–42.0)	35	40.7 (29.8–51.7)	0.08	–	–	–
Outcome variables (continuous)	Number of Unhealthy Gambling Indicators^b^						Phase ONE		Phase TWO	
	None		One		Two or More		p-value^c^	Risk, protective, non-sig (−)	p-value^c^	Risk, protective, non-sig (−)
	n (1,849)	Median (95 % CI)	n (1,849)	Median (95 % CI)	n (1,849)	Median (95 % CI)				
Social connectedness:										
Family	1,557	0.1 (−1.3-0.9)	203	−0.1 (−1.3-0.9)	89	−0.2 (−1.8-0.7)	<.0001	Protective	0.28	–
Friends	1,557	0.3 (−1.5–0.6)	203	0.3 (−1.3–0.6)	89	−0.0 (−1.6–0.6)	0.02	–	–	–
School	1,557	−0.0 (−1.00–0.8)	203	0.0 (−0.8–0.8)	89	−0.1 (−1.4–1.0)	0.01	–	–	–

^a^Amongst students who had gambled in the past 12 months

^b^Based on the construct of unhealthy gambling behaviour developed as part of this study

^c^Analyses have controlled for sex, age, socioeconomic deprivation, geography (urban/rural), and ethnicity

Multiple logistic regressions between the measure of ‘unhealthy gambling’ and the variables that fulfilled significant risk/protective functions were carried out to determine if these items maintained their risk/protective functions in the presence of each other (phase two analyses). As detailed in Tables 3 and 4, four variables were associated with an increased risk of ‘unhealthy gambling’ in Phase two, specifically: worried about and/or had tried to cut down on their gambling (OR 2.6 and OR 5.1); more accepting attitudes towards gambling (OR 4.2); gambled on EGMs, casinos, or horse/dog/sports (‘TAB’) betting in the past 12 months (OR 3.8 and OR 6.7); and, had attempted suicide in the past 12 months (OR 4.0 and OR 7.9). None of the investigated protective items continued to maintain a measureable protective role.

**Table 4:** Logistic regression models - odds ratios for significant risk factors as identified in Phase Two^a^

Outcome variables (categorical)	Number of Unhealthy Gambling Indicators^b^		
	None	One	Two or more
	Odds ratio (95 % CI)	Odds ratio (95 % CI)	Odds ratio (95 % CI)
Activities that student has gambled on (last 12 months):			
EGMs/Casino/TAB betting	1	3.8 (2.6–5.5)	6.7 (3.7–12.1)
Attitudes and social modelling of gambling:			
More accepting of gambling	1	1.2 (0.8–1.9)	4.2 (2.1–8.6)
Concerns about gambling:			
Worried/cut-down	1	2.6 (1.7–4.0)	5.1 (3.0–8.6)
Emotional health and wellbeing:			
Suicide attempt	1	4.0 (2.3–7.2)	7.9 (4.2–14.9)

^a^Amongst students who had gambled in the past 12 months

^b^Based on the construct of unhealthy gambling behaviour developed as part of this study

## Discussion

### Summary of Main Findings

Most students in this study had limited engagement with gambling activities. However, substantial proportions of students who had gambled in the past year reported factors that were indicative of unhealthy gambling, with these rates being comparable to those observed in the Youth’07 study (Rossen et al. 2011). Males, students from Māori, Pacific and Asian communities, those living in highly socio-economically deprived neighbourhoods, urban students and same/both-sex or not-sure attracted young people (i.e. sexual minority youth) are disproportionately at risk. Having more accepting attitudes towards gambling; gambling on EGMs, casinos, or horse/dog/sports betting in the past 12 months; being worried about and/or had tried to cut down on their gambling; and, attempting suicide in the past 12 months were associated with a higher risk of unhealthy gambling among a representative sample of secondary school students.

This research explored associations between a number of variables hypothesised to be a risk and/or protective factor of unhealthy gambling. The importance of supportive family environments was highlighted in Phase one of the analyses which indicated that family connectedness acted in a protective manner, whilst the family impacts of someone else’s gambling (i.e. arguments in family, had to go without, bills not paid and dishonest acts) were all risk factors. The multivariate model suggests that while family connection acted in a protective manner, once other factors are accounted for in the model; it would appear that exposure to adult gambling behaviours and mental health indicators are more critical. These findings emphasise the need to consider family dynamics and support networks, and the importance of assessing the impacts of parental gambling on the children of adult clients with gambling-related issues when addressing the issue of unhealthy youth gambling (Clarke, Abbott, DeSouza, and Bellringer 2007).

The significant relationships between unhealthy gambling and other emotional/mental health issues are important indicators for clinicians, educators and policy makers to address. Students with unhealthy gambling practices were significantly more likely to report co-existing mental health issues (e.g. depression and suicide attempts) and other addictions/risky behaviours (e.g. use of alcohol and weekly cigarette smoking) emphasising the need for clinicians to consider unhealthy gambling alongside other health issues. The findings are consistent with Shead et al. (2010) who argued that many of the risk factors for unhealthy youth gambling predict a general behaviour syndrome that is encompassed by overall mental health problems. This aligns with research that has shown a tendency for clinically concerning health risk and emotional health concerns to cluster in young people (Noel et al. 2013). The authors argue that this demonstrates a need for comprehensive psychosocial assessments and the provision of appropriate services, particularly for ‘at-risk’ youth.

Moreover, the finding that young people with more accepting attitudes towards gambling are at greater risk of problems has implications for health promotion efforts. Social acceptability (by parents and peers) is recognised as an important socio-ecological driver of other risky behaviours in youth, including marijuana and illicit drug use, binge drinking and smoking (Bahr, Hoffmann, and Yang 2005; Simons-Morton and Farhat 2010). Efforts to adjust youth attitudes towards gambling will have to reflect an ecological approach that considers socialisation processes and takes into account the attitudes of parents, peers and society at large.

This research identified a number of important disparities based on demographic features, largely consistent with previous gambling research involving young people from New Zealand (Devlin 2011; Health Sponsorship Council 2012; Rossen et al. 2011). Consistent with research from Greece and Finland (Floros et al. 2013; Raisamo, Halme, Murto, and Lintonen 2013), gambling appears to play a more prominent role in the life of young males than females in New Zealand. Overall, the disproportionately higher percentages of youth gambling and/or unhealthy gambling behaviour amongst Māori, Pacific and Asian students, and students living in highly deprived neighbourhoods, corresponds with findings from adult-based gambling research in New Zealand (Abbott, Bellringer, Garrett, and Mundy-McPherson 2014; Abbott and Volberg 2000; Bellringer, Perese, Abbott, and Williams 2006; Devlin 2011; Perese, Bellringer, Williams, and Abbott 2009; Tu'itahi, Guttenbeil-Po'uhila, Hand, and Htay 2004). Sexual minority students were another population with a heightened risk of problematic gambling. These youth were significantly more likely to report unhealthy gambling compared to their exclusively opposite-sex attracted peers. To the authors’ knowledge, this finding is unique; while sexuality and gambling has been explored in adults (Liao 2014) it has not previously been explored in relation to youth gambling. This finding does however correspond with prior research on substance use, where youth that were not exclusively opposite-sex attracted were, on average, 190 % more likely to report substance use than heterosexual youth (Marshal et al. 2008). Specific strategies are required that address ethnic, sexuality and social disparities faced by certain populations vulnerable to unhealthy gambling.

Young people rarely seek formal help for gambling-related problems (Griffiths 2001; Gupta and Derevensky 2000). Nonetheless, research from Australia and North America has indicated that negative emotional states such as stress, depression and guilt are strong motivators for help-seeking amongst adults (Evans and Delfabbro 2005; Hodgins & el-Guebaly 2000), and research with New Zealand adults has shown that a lack of self-awareness (i.e. denial of gambling problems) is a barrier to help-seeking (Clarke et al. 2007). Of the students who were gambling at unhealthy levels in this study, more than half demonstrated self-awareness (i.e. they were worried about or had tried to cut down on their gambling). This self-awareness may be an important indicator that an appropriate opportunity exists to motivate a young person to address their gambling through a suitable intervention.

The host responsibility of gambling venues in New Zealand requires urgent improvement; despite legislation prohibiting minors from gambling on EGMs, casinos, and horse/dog/sports (‘TAB’) betting, significant proportions of students reported engaging in these intense forms of gambling. Moreover, involvement in these modes was associated with a high risk of unhealthy gambling – consistent with previous research highlighting the dangers of continuous modes of gambling (i.e. those with continuous/immediate feedback cycles) (Abbott and Volberg 2000; Health Sponsorship Council 2012).

### Strengths and Limitations

The data presented in this study are from a large nationally representative sample of secondary school students in New Zealand. This study utilised a unique non-dichotomous measure of problematic gambling which is advantageous as it reflects the more fluid nature of youth behavioural issues.

There are a number of limitations to the study. Young people who are disengaged from a mainstream school environment (e.g. students in alternative education programmes outside of mainstream schooling and young people who have ‘dropped out’ of school) are not represented in these findings. Moreover, only students who were at school on the day of the survey were included. Therefore the findings outlined in this paper may represent a more ‘positive’ view of gambling-related issues for New Zealand’s youth, as evidence suggests that young people who are disengaged from mainstream education are not as healthy as their peers (Bovet, Viswanathan, Faeh, and Warren 2006; Clark et al. 2010; Denny, Clark, and Watson 2004). Additionally, the Youth’12 survey is a cross-sectional survey, and as such it is important to note that while a number of relationships/associations have been observed between variables, these are not necessarily indicative of cause and effect, and directionality cannot be determined. For instance, it is unclear if problematic gambling leads to associated financial and other issues, which then lead to depression or if gambling is driven by a pre-existing mental health issue.

## Conclusions

Unhealthy gambling impacts on a numerically small group, but is significantly problematic amongst certain New Zealand secondary school students and their families. Youth affected by unhealthy gambling are likely to have other social, addiction and emotional/mental health challenges.
